# Transparent Ultraviolet (UV)-Shielding Films Made from Waste Hemp Hurd and Polyvinyl Alcohol (PVA)

**DOI:** 10.3390/polym12051190

**Published:** 2020-05-22

**Authors:** Yi Zhang, Rechana Remadevi, Juan P. Hinestroza, Xungai Wang, Maryam Naebe

**Affiliations:** 1 Institute for Frontier Materials, Deakin University, 75 Pigdons Road, Geelong, Victoria 3216, Australia; ivf@deakin.edu.au (Y.Z.); rechana.remadevi@deakin.edu.au (R.R.); xungai.wang@deakin.edu.au (X.W.); 2 Fiber Science and Apparel Design, Cornell University, Ithaca, NY 14853, USA; jh433@cornell.edu

**Keywords:** lignin, hemp hurd waste, UV-shielding, mechanical property, PVA, ball milling, lignocellulosic particles, plastic film

## Abstract

This work proposes a new approach to fabricate highly transparent and flexible composite films that exhibit enhanced UV-shielding properties. Lignin has innate UV-shielding properties. However, when purified lignin, which is conventionally extracted through chemical treatment, is mixed with polymeric materials, its presence negatively influences the transparency of the resulting composite. High transparency and UV-shielding are desirable properties for many applications. In this study, composites were made by mixing lignocellulose particles and polyvinyl alcohol (PVA), where lignocellulose particles were obtained from ball-milled waste hemp hurd without chemical treatments. The UV-shielding properties of the resulting composite film, as a function of hemp/PVA weight ratios, were investigated. The intermolecular interactions between the hemp particles and the PVA were characterized using infrared spectroscopy with the presence of –C=O group at 1655 cm^−1^, providing evidence that the chemical structure of lignin was preserved. The fabricated hemp/PVA films exhibit stronger UV-shielding, in the UVA-I range (340–400 nm) than TiO_2_/PVA films. The composite films also showed comparable water vapor permeability (WVP) with commercial packaging plastic film made of HDPE (high-density polyethylene). The optimization experiments were reported, with aim at understanding the balance between the UV-shielding and mechanical properties of the hemp/PVA films. The findings of this work can be applicable to the packaging, food and cosmetic industries where UV shielding is of utmost importance, hence adding value to hemp hurd waste.

## 1. Introduction

Long-term exposure to ultraviolet radiation causes severe damage to animals, plants and to some materials [1]. UV shielding research has focused mainly on the role of inorganic metal oxides such as titanium dioxide (TiO_2_), zinc dioxide (ZnO) and silicon dioxide (SiO_2_) [2,3,4]. Although inorganic metal oxides exhibit good UV-shielding, their synthesis and disposal methods may have some environmental and health concerns [5]. Furthermore, inorganic metal oxides have strong photocatalytic activity which accelerate the photo-degradation of polymer matrices [6], hence limiting their use in food packaging, cosmetics and pharmaceuticals. 

Lignin has innate UV-shielding properties and it has been proposed as a substitute for inorganic metal oxides [7]. Use of lignin as a UV-shielding additive may reduce the environmental and health impacts of inorganic metal oxides [5]. Research work on the UV shielding properties of lignin has been focused on understanding the influence of its size [8], chemical modifications [1] and its sourcing [9].

Studies on lignin particles showed that nanoparticles exhibit better UV-shielding properties than micron-sized particles [8] as lignin nanoparticles have larger surface areas and higher transparencies. Qian et al. [9] used alkali to extract lignin and used it in a natural broad-spectrum sunscreen. However, the addition of lignin reduced the transparency of the resulting sunscreen cream [10]. High transparency is required by many applications such as optoelectronics [11], food packaging [12] and solar energy [13]. Xiong et al. [14] increased the transparency of a fabricated lignin/PVA composite by reducing the amount of lignin nanoparticles to 3 wt % via enzymatic hydrolysis using tetrahydrofuran, a highly toxic solvent. 

Lignin, cellulose and hemicellulose are the three main components of lignocellulose materials [15]. The lignin present in lignocellulose is pale-yellow, compared to the dark colour of alkali-extracted lignin. This lighter colour implies that lignocellulose can be used as a filler in UV-shielding products, that is, wood powder has already been added to sunscreen [16]. Hemp is a fast-growing plant that consists of fibers and hurd. Hemp hurd, the woody part of hemp, is a low-cost by-product from the decortication process, the process that removes the hurd from the softer and fibrous exterior of the stalk. Hemp hurd, is a rich source of lignocellulose and it is composed of cellulose (40 wt %–48 wt %) and lignin (21 wt %–24 wt %) [17,18]. Compared with other agricultural waste such as triticale straw and corn residue, lignin in hemp hurd contains a high amount of total phenolic groups and syringyl phenolic groups which exhibit UV-shielding properties [19]. While the retaining price of hemp hurd is lower than $0.2/kg, 70–80% of hemp hurd is wasted and commonly disposed via combustion or landfilling [20]. Thus, using hemp hurd as UV-shielding materials would be cost effective as the syringyl lignin and total phenolic group per g are higher than other agricultural waste.

Polyvinyl alcohol (PVA) is a colourless, biocompatible and biodegradable, non-toxic and water-soluble semicrystalline synthetic polymer and it possesses unique mechanical and thermal properties that make it amenable to film-forming processes [21]. PVA-based composites are used in food packaging films, tissue scaffolding, enzyme immobilization substrates and drug release applications [22]. PVA has low UV shielding properties and it degrades under UV light [23].

The objective of this study was to investigate the UV shielding of lignocellulose hemp particles on a hemp/PVA composite film fabricated through a sustainable approach. Hemp hurd, a readily available waste from the hemp industry, was used and lignocellulose particles of hemp were obtained via a wet ball-milling method, without using any chemical treatment. Films were prepared using a one-step casting method, with the lignocellulose particles from the hemp hurd used as reinforcement and PVA as a polymer matrix. The morphology of the hemp hurd particles as well as the mechanical, transparency and UV shielding properties of the composite films were investigated. 

## 2. Materials and Methods 

### 2.1. Materials

Hemp hurd (Australian grown), weighed 200 g, was sourced from the Commonwealth Scientific and Industrial Research Organisation (CSIRO, Waurn Pounds). The sample was dried at 60 °C for 12 h, followed by chopping off into coarse particles with a Fritsch Pulverisette 19 Universal Cutter Mill (sieve diameter of 200 µm). Polyvinyl alcohol solution (PVA) (5 wt %, with a viscosity of 100–120 mPas, hydrolysis degree of 87–89%) was purchased from Flew solutions (Queensland, Australia).

### 2.2. Fabrication of Particles

Fifty grams of coarse hemp hurd particles were mixed with 2 L of deionized water to produce a hemp slurry. The slurry was wet ball-milled with an Attritor mill (2S, Union Process, Akron, OH, USA) and an agitator speed of 280 rpm for 4 h and 20 h. 

### 2.3. Preparation of Hemp Hurd/PVA Film

The casting solution was composed of the 20 h ball-milled slurry (hemp in water) and the PVA solution. The casting solutions were prepared with different weight percentages of hemp particles (0(w/w) %, 1(w/w) %, 5(w/w) % and 10(w/w) %). The film samples were named as “pure PVA,” “1 wt. % film,” “5 wt. % film” and “10 wt. % film.” For all specimens 6.6 g of PVA solution were used. After drying for 24 h in a fume hood at room temperature, a film with a diameter of 84 cm and 40 µm thickness was obtained. Care was taken to avoid the influence of the residual water content and maintain similar hydration degree for films; therefore, the film samples were dried in oven for 30 min at 30 °C before testing and were then kept in a sealed plastic bag.

### 2.4. Material Characterisation 

#### 2.4.1. Particle Size Analysis 

Hemp particles were obtained through 4 h, 8 h, 12 h, 16 h and 20 h ball of milling and sonicated for 5 min to prevent agglomeration. The particle size of the samples was measured using a dynamic light scattering method [ISO22412] on a Zetasizer (Malvern, UK). The size of the cutter-milled particles was measured with a Mastersizer analyzer (2000, Malvern, UK).

#### 2.4.2. Scanning Electron Microscopy (SEM)

Cross-sections of the film were prepared using the liquid nitrogen fracturing method. The morphology of the hemp particles, the cross-section and surface of the film was observed using a secondary electron (SE2) detector in a SEM (Zeiss Supra 55 VP, EV = 5 kV). Prior to imaging, the samples were gold coated using a sputter coater (BAL-TEC SCD 050, Leica, 5 nm). 

#### 2.4.3. X-ray Diffraction (XRD)

The slurries produced using the 4 h and 20 h ball milling were dried in a spray dry machine (B-290, Buchi Labortechnik AG, Switzerland) to produce dried particles for XRD characterization. The intensity in the 2θ range from 6° to 40° obtained in the XRD (Panalytical, Almelo, Netherlands) were used to calculate the Crystallinity index (CrI) of the samples according to Equation (1)

(1) $$\mathrm{CrI}\;=\frac{I_{002}-I_{\mathrm{am}}}{I_{002}}\times 100\%,$$

where I_002_ is the diffraction intensity of 2θ located around 22.5° and corresponding to the crystalline domain. I_am_ is the diffraction intensity of 2θ, located around 18° and corresponding to the amorphous domain [24].

The crystallinity of the film was calculated by Equation (2)

(2) $$X_{c}=\frac{A_{c}}{A_{c}+A_{a}},$$

where $X_{c}$ is the crystalline fraction, $A_{c}$ represents the crystalline area and $A_{a}$ represents the amorphous area. The crystalline and the amorphous areas were obtained through the deconvolution method [24]. 

#### 2.4.4. Fourier Transform Infrared (FTIR) Spectroscopy

The chemical structure of the hemp/PVA composite was studied using a Bruker Vertex spectrometer (Massachusetts, USA) with an Attenuated total reflection (ATR) at a resolution of 4 cm^−1^.

#### 2.4.5. Mechanical Properties of the Films

Tensile tests were conducted using a universal tensile testing machine (Instron, USA) at a temperature of 20 ±  2 °C and a relative humidity of 62  ±  2%. Samples (30 mm in length × 10 mm in width) were cut according to ASTM D882 [25]. The gauge length $(l_{0}$) of the sample was 10 mm. The tensile rate was kept at 4 mm/min, the load was recorded with a 100 N load cell and the extension Δl was measured with an extensometer. The first linear part of the stress-strain curve was used to calculate Young’s modulus. The tensile strength was represented by the ultimate tensile stress, which equals to the load divided by the cross-section area (0.004 mm^2^) of the film.

#### 2.4.6. Water Vapor Permeability 

The water vapor permeability (WVP) of hemp/PVA composite films was measured with a Moisture Vapor Transmission Rate Tester (Labthink’s W3/031, China). The samples were measured at 90% relative humidity and 38 °C according to TAPPI T 464 [26].

#### 2.4.7. UV-Shielding 

A UV-Vis-NIR spectrophotometer (Cary 5000 Scan, Varian Inc., Palo Alto, CA, USA) was used to test the transmittance of the film with a scan rate of 600 nm/min. The wavelength of the light beam ranged from 200 nm to 800 nm. The ultraviolet protection factor (UPF), used to represent the UV-shielding performance of the films, was obtained with a YG902 UPF spectrophotometer according to the AS/NZS 4399 standard. The UV-shielding properties of the films were normalized to UPF values (UPF value per weight).

#### 2.4.8. Statistical Analysis

A one-way ANOVA test, run with SPSS Statistics software, Version 26, 2019, was used to analyze the significance of the difference between the four films, where *p* ≥ 0.05 indicates no significant differences between the datasets and *p* ≤ 0.05 indicates that there is a significant difference between the data.

## 3. Results and Discussion

### 3.1. Particle Size Analysis 

**Figure 1:**
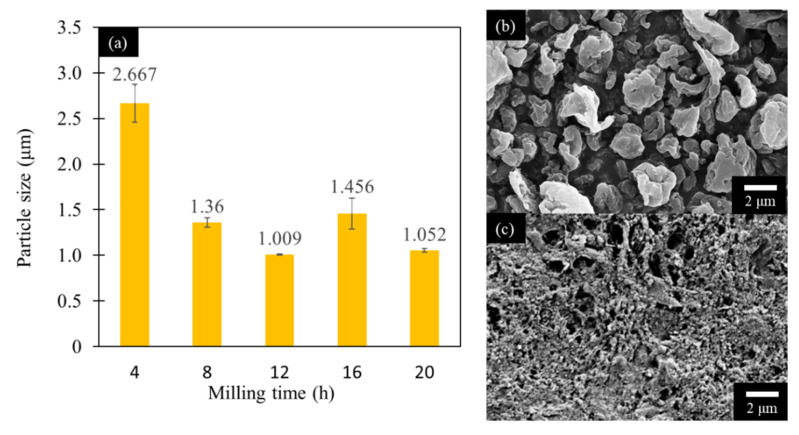
(**a**) Particle size as a function of wet milling time; scanning electron microscope (SEM) images of hemp particles: (**b**) after 4 h and (**c**) after 20 h of wet ball-milling.

The De Brouckere mean diameter, D [3,4] of the cutter milled hemp hurd particles was measured at 253.5 μm. The Z-average size as a function of milling time is shown in Figure 1a. A 50% reduction in mean particle size was observed as the milling time increased from 4 h to 8 h. After 8 h, the particle size remained around 1 μm indicating the size limit for the ball milling of hemp. After 16 h of milling time, the mean particle size increased from 1 μm to 1.46 μm, suggesting aggregation or agglomeration. 

To further understand the effect of milling time on the morphology of hemp, SEM images were used as shown in Figure 1b,c. It was observed that in the sample with 4 h wet ball-milling, the majority of the hemp crushed into particles of irregular shape (Figure 1b). As the milling time increased to 20 h, the irregular particles were split and fractured, contributing to more uniform and smaller particles (Figure 1c).

### 3.2. XRD Analysis 

**Figure 2:**
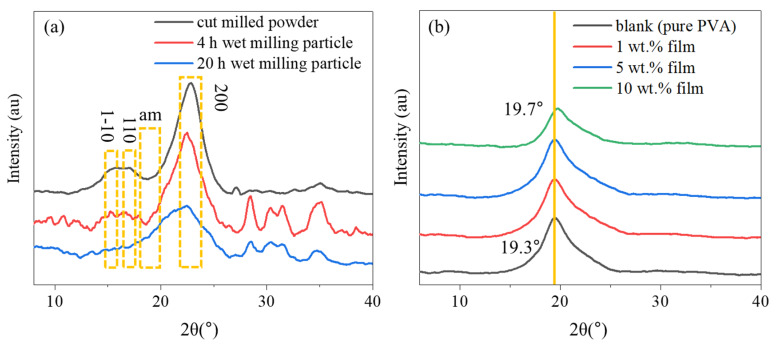
X-ray diffraction (XRD) pattern of (**a**) hemp particles and (**b**) composite films.

Figure 2a shows the XRD pattern of the hemp particles. Three main diffraction peaks at 2θ around 14.5°, 16.5° and 22.5° corresponding to the cellulose-I crystal’s planes of (1–10), (110), (200) [27,28] were observed. Table 1 shows that the CrI of the hemp particles reduced only 2% after 4 h but reduced 35% after 20 h of wet ball-milling. The reduction of CrI indicates that the mechanical force generated from the ceramic balls and impellers during the wet ball-milling damaged the ordered structure of particles [29]. When size reduction reached the particle limit of the milling device, the continued mechanical energy transferred from the mill to the hemp causes the accumulation of defects on the crystal structure of hemp and the disorder of the crystal, leading to the reduction of crystallinity [30]. During the ball milling procedure, the intensity of the crystal’s planes of (200) and (110) corresponded to the crystallites diameter and the directions perpendicular to crystallites [30]. 

Hemp particles (after 20 h of milling) were added to the PVA solutions at 1%, 5% and 10% weight ratios. Figure 2b shows the XRD patterns of the pure PVA and the hemp/PVA films. The crystallinity (Xc) of the film was also calculated using Equation (2). A representative of the deconvolution of the pure PVA film as an example has been provided in Figure S1. The crystallinity of pure PVA, 1% film, 5% film and 10% film were 21.41%, 23.89%, 23.89% and 42.20% respectively. As shown in Figure 2b, the main diffraction peak of the pure PVA at 19.3° is related to the (101) γ-crystalline phase of PVA [11]. The variation in this peak after loading with 1% and 5% hemp suggests that the hemp particle stabilized the nature of PVA [31]. While adding 10 wt. % of hemp particles, the 2θ of the peak for the film shifted from 19.3° to 19.7°, indicating that the structure of pure PVA has been influenced by adding more particles. The peak shifting to a higher degree suggests that the lattice structure of 10% film changed probably due to the compositional impurities in the crystal structure [32] and this might be the reason for higher CrI of the 10% film compared to the 1% and 5% films. In addition, hemp particles showed higher crystallinity (46.96%, Table 1) than PVA film (21.41%) and crystallinity of the composite film was influenced by the amount of hemp particle. Loading PVA film with the higher amount of hemp particles (10%) with high CrI resulted in film with higher CrI compared to the pure PVA film. 

### 3.3. Morphology of the Films

**Figure 3:**
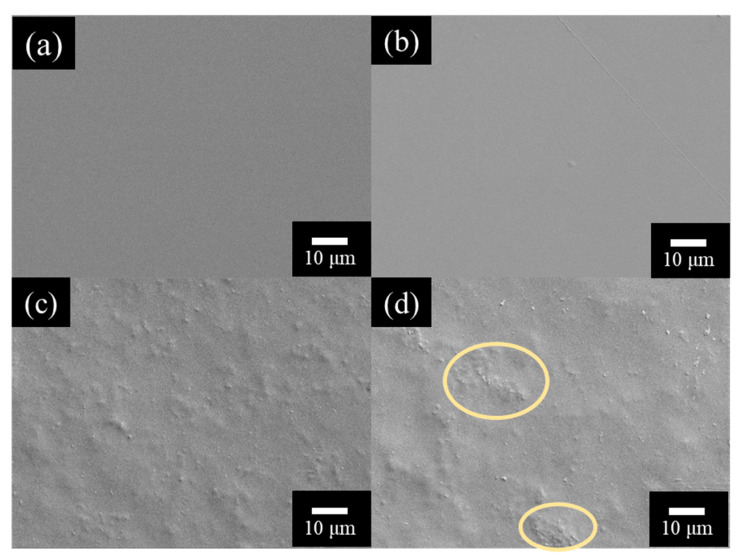
The surface of hemp/PVA composite films with different hemp particle weight ratios (**a**) 0%, (**b**) 1%, (**c**) 5%, (**d**) 10%.

The surface morphology of pure PVA and the hemp/PVA films are shown in Figure 3. Hemp particles appear to the microstructure of the film. The smooth surfaces of the pure PVA film and the hemp /PVA composite film (1 wt. %) were relatively identical. The surface of the films with 5 wt. % and 10 wt. % of hemp particles appeared rougher and clusters were observed in the 10 wt. % film which indicated particles agglomeration.

**Figure 4:**
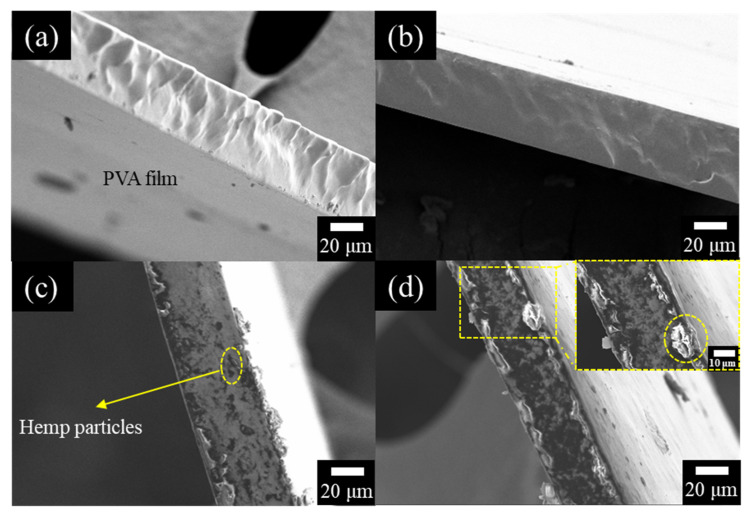
The cross-section images of hemp/ polyvinyl alcohol (PVA) composite films with different hemp particle weight ratios: (**a**) 0%, (**b**) 1%, (**c**) 5%, (**d**) 10%.

The SEM images of the cross-section of the films are shown in Figure 4. As shown in Figure 4a,b, the cross-section of the pure PVA film and 1 wt. % hemp/PVA film appears relatively smooth and homogeneous, maintaining a relatively continuous phase. The 5 wt. % hemp/PVA film shows some particles irregularly embedded in the film, as shown in Figure 4c. The 10 wt. % hemp/PVA film illustrated a denser structure with hemp particles, due to the higher amount of particles added to the film. The film’s cross-section (Figure 4d) shows large hemp particles on the border edge which further confirmed the aggregation and clustering of the hemp particles as shown in Figure 3. The aggregation also slightly changed the thickness and uniformity of the edges. 

### 3.4. ATR-FTIR Analysis of the Films 

**Figure 5:**
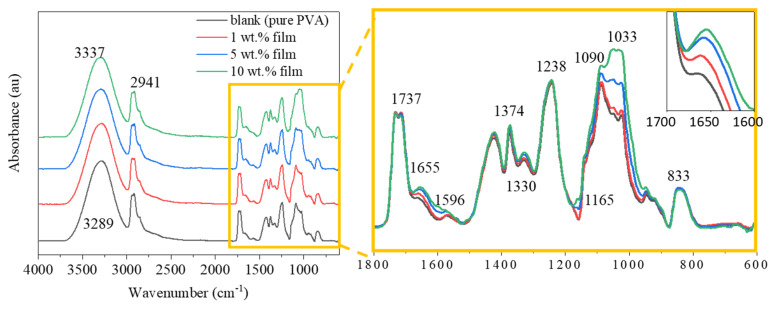
Fourier Transform Infrared (FTIR) spectra of pure PVA and hemp/PVA composite films.

Figure 5 shows the FTIR spectra of the films. Since a maximum 10% of hemp particles was mixed with PVA and large proportion of films were made of PVA, most of the peaks were corresponded to the PVA structure. However, the main peaks related to the hemp, cellulose, hemicellulose and lignin were also detected along with the corresponding PVA peaks.

As shown in Figure 5, the absorption peak observed at 3289 cm^−1^ is attributed to the -OH stretching vibration of inter- and intra-molecular hydrogen bonds [15,33]. With increasing the amount of hemp powder, the peak at 3289 cm^−1^ shifted to a higher wavenumber of 3337 cm^−1^. This might be due to the formation of hydrogen bonds in the composite film and the higher amount of hemp means more hydrogen bonds between hemp and PVA. The –OH groups of the lignin are capable of forming hydrogen bonds with the PVA polymer matrix [34]. The infrared spectra at 2941 cm^−1^, 1238 cm^−1^ and 833 cm^−1^ are the typical peaks of C-H [35] from cellulose, hemicellulose and lignin. The peak at 1737 cm^−1^ represents the –C=O bonds in xylan from hemicellulose [36]. The peak at 1655 cm^−1^ and 1596 cm^−1^ are the vibrations of the –C=O in quinone or p-quinone and C=C in the aromatic structure which only exist in lignin, indicating that lignin has been persevered in hemp particle. The peak at 1374 cm^−1^ represented symmetric and asymmetric bending of CH_3_ groups, which were from cellulose and lignin. The peak at 1330 cm^−1^ resulted from the –C–O stretching of the syringyl ring from lignin. The band at 1033 cm^−1^, 1090 cm^−1^ and 1165 cm^−1^ is due to C-O stretching [33] which is assigned as cellulose and hemicellulose.

### 3.5. Mechanical Properties of the Film 

**Figure 6:**
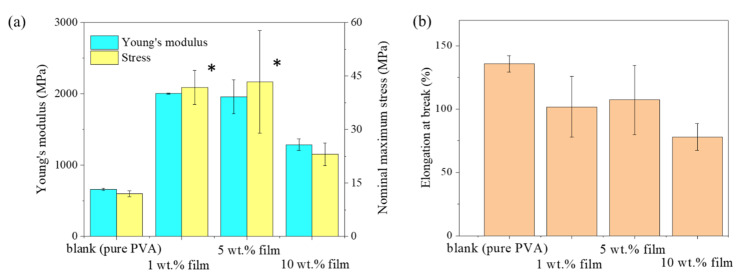
Mechanical properties of pure PVA and hemp/PVA composite films: (**a**) Young’s modulus and nominal maximum stress (**b**) Elongation at break (%). * denotes significant difference (*p* ≤ 0.05) was found between data.

Figure 6 shows the Young’s modulus and strength of the hemp/PVA composite films and their statistical analysis of the results (Table 2). The elongation at break for the composite films was lower than the elongation at break for the pure PVA film. The 1 wt. % and 5 wt. % hemp/PVA composite films showed a significant increase (*p* ≤ 0.05, Table 2) in Young’s modulus, almost 4 times higher than the Young Modulus for the pure PVA film. The same trend was observed for strength. The crystallinity of the 1 wt. % and 5 wt. % film were higher than pure PVA, as confirmed by the XRD result, resulted in increased Young’s Modulus [37]. With the further addition of hemp particles to 10 wt. %, both Young’s modulus and nominal maximum stress (tensile load divided by the area of initial cross-section) decreased but still were higher than those of the pure PVA film. Because nonhomogeneous coarse particles were present in the film (as shown in Figure 4), Young’s modulus and nominal stress deteriorate with higher particle concentration (e.g., 10% wt. %) [38]. In addition, the formation of inter-hydrogen molecular bonds (peak change from 3289 cm^−1^ to 3337 cm^−1^) between PVA and –OH groups may contribute to the increase of Young’s modulus and stress of 1 wt. % film, 5 wt. % film and 10 wt. % film compared with pure PVA. The structure of the film was likely influenced by the amount of hemp particles and with increasing the number of hemp particles, particle clusters were found in SEM results. The reduction in Young’s modulus and nominal maximum stress of 10 wt. % might be due to aggregation of the particle in the composite film.

With the adding of hemp particles, the elongation at break of all PVA composite films decreased compared to pure PVA. However, the changes in elongation were not significant (*p* ≥ 0.05, Table 2) among all composite films, indicating that elongation of the films was not influenced by the amount of hemp particle. All fabricated films were also highly flexible and foldable. 

### 3.6. Water Vapour Permeability (WVP) Properties of Hemp/PVA Composites

**Figure 7:**
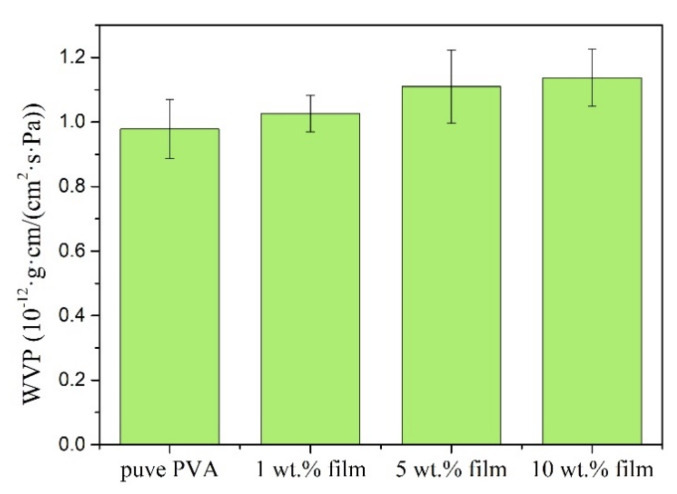
Water vapor permeability (WVP) of hemp/PVA composite films versus hemp loadings.

Figure 7 shows the effect of hemp content on the water vapor permeability of hemp/PVA composites. The film made from pure PVA exhibits a low water vapor permeability of 0.98 × 10^−12^ g·cm/(cm^2^·s·Pa). When the amount of hemp reached 10%, the WVP of the composite films reached a maximum of 1.14 × 10^−12^ g·cm/(cm^2^·s·Pa). The films became soft due to the water absorption [39] and this absorption may have an influence in the hydrogen bonding between hemp and PVA [40]. However, the differences between WVP of the composite films were not found to be significant (*p* > 0.05). The WVP value of the commercial packaging plastic film made of HDPE (High-density polyethylene) at 37.8 °C and 90% RH has been reported to be 1.741–3.482 × 10^−12^ g·cm/(cm^2^·s·Pa) [41]. This indicates the fabricated hemp/PVA composite film in this study has a strong potential for food packaging applications.

### 3.7. UV-Shielding Performance of the Film

**Figure 8:**
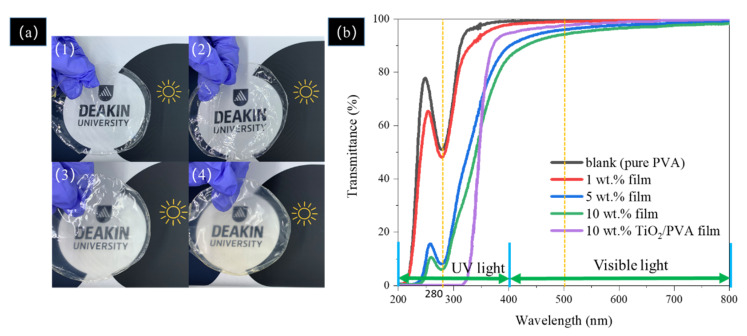
(**a**) Photographs of the film: (1) pure PVA, (2) 1 wt. % film, (3) 5 wt. % film, (4)10 wt. % film; (**b**) Transmission spectra of the composite films with various hemp hurd additions in this study and TiO_2_/PVA film reproduced from Liu et al. [42].

Figure 8a shows photographs of the PVA film and PVA composite films fabricated with hemp particles after 20 h ball milling and Figure 8b shows the optical properties of the film under UV (200–400 nm) and visible (400–800 nm) light. The wavelengths of 280 nm and 500 nm were selected for comparison of the sample’s performance under UV and visible light. In visible light at 500 nm, the pure PVA has a transmittance of 99.5% and the film with 10 wt. % of hemp particles has a transmittance of 94.3%. 

The pure PVA film 100% blocked UV light in a very narrow range of 200–212 nm, while the films with 5 wt. % and 10 wt. % showed UV blocking properties in a broader range of 200 nm to 231 nm. At 280 nm, the transmittance of the pure PVA was 51%, while the transmittance for the films loaded with 1 wt. %, 5 wt. % and 10 wt. % of hemp particles was 48%, 8% and 6%, respectively. Liu et al. [42] manufactured a PVA film with 10% of TiO_2_ that showed 100% blocking at the wavelength of 280 nm and a transmittance of 98% at the wavelength of 500 nm. TiO_2_ nanoparticles showed photocatalytic effects that can degrade the polymer. The PVA films loaded with 5 wt. % film and 10 wt. % of hemp showed better performance at the wavelength range of 345–400 nm when compared to the 10 wt. % TiO_2_/PVA film. The 90% of UV radiation reaching the Earth’s surface is UVA (315–400 nm). Exposure to UVA-I damages human dermal fibroblasts [43]. 

**Figure 9:**
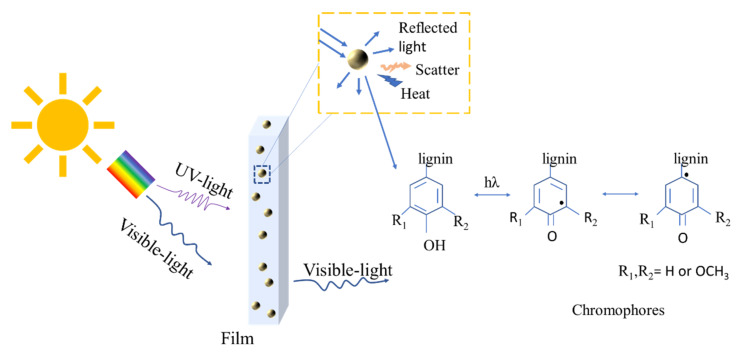
The proposed mechanism of UV shielding performance of hemp/PVA film.

It is known that the chromophore group of –C=O is capable of absorbing UV-light [44], therefore, it can be concluded that the UV blocking properties of the hemp/PVA films are due to the presence of –C=O groups in quinone or p-quinone of lignin. When the UV light reaches hemp particles, the photon energy of UV light is converted to heat with the corresponding hydrophilic chromophores, mainly phenolic hydroxyl, carbonyl and carboxyl groups [10]. The phenolic hydroxyl groups of lignin absorb the energy from UV radiation, which could also easily quench active radicals (Figure 9) through an electron transfer process. Then, the generated heat is gradually released out of the composite films.

The normalised UPF values shown in Table 3 are presented to consider the different weights of the fabricated films. While the UPF value of 1 wt. % film was as low as that of pure PVA, the UPF value of the film loaded with 5 wt. % of hemp particles was almost 7 times higher than pure PVA. The 10 wt. % film showed the highest UPF value, 16 times higher than that of the pure PVA.

## 4. Conclusions

In this study, UV-shielding and transparency were achieved in flexible hemp/PVA films through the use of lignocellulose from hemp hurd. Lignocellulose particles were prepared through a wet ball milling method without chemical treatments. To understand the limitations of particle size, the relation between milling time and particle size was studied. The smallest size of particles was around 1 µm and crystallinity of the particles reduced after 20 h wet ball milling. SEM image shows the homogenized distribution of the micro-particles in the film with low additive amounts. While all fabricated films were highly flexible, the 1 wt. % and 5 wt. % hemp/PVA composite films showed a significant increase (*p* ≤ 0.05) in Young’s modulus and nominal maximum stress, which were considerably higher than pure PVA film. The hydrogen bond between hemp and PVA and an increase in the crystallinity of film, maybe the reason for enhanced Young’s modulus and higher nominal maximum stress of the film. The decrease in Young’s modulus and in nominal maximum stress of the 10 wt. % hemp/PVA film were caused by the hemp particle aggregation, as confirmed by SEM. Compared to a previously fabricated TiO_2_/PVA film, the hemp/PVA composite films showed better UV shielding properties performance in the UVA-I range which is a critical range damaging human skin cell. The highest UV-shielding ability of the film was achieved with 10 wt. % hemp. The FTIR results also proved the existing of –C=O groups in quinone or p-quinone of lignin that were persevered by ball milling method. The 5 wt. % film exhibits a good balance between mechanical properties and UV-shielding properties with highly transparent optical properties. The water vapor permeability (WVP) of the composite films showed higher WVP compared to the pure PVA film and comparable with the commercial packaging plastic film made of HDPE (High-density polyethylene). This work provides additional value to agriculture hemp waste enabling its used as UV-shielding additive. 

## Figures and Tables

**Table 1:** CrI of hemp particles after different treatment.

	CrI (%)	I_200_	I_am_
cutter milled	84.26	0.98759	0.1554
4 h wet ball-milling	81.97	0.90891	0.16388
20 h wet ball-milling	46.96	0.56037	0.29723

**Table 2:** Statistical indicator: *p*-value * of multiple comparisons of films’ mechanical properties.

		Young’s Modulus	Nominal Maximum Stress	Elongation at Break (%)
			*p*-Value	
pure PVA	1 wt. % film	0.033	0.027	0.318
	5 wt. % film	0.037	0.021	0.397
	10 wt. % film	0.057	0.348	0.108

* p ≤ 0.05 indicates that there is a significant difference between the data.

**Table 3:** Ultraviolet protection factor of the films (UPF).

	UPF	Normalised UPF	Protection Grade
PVA film	2.03	6.15	poor
1 wt. % film	2.76	8.28	poor
5 wt. % film	15.07	43.39	good
10 wt. % film	35.79	98.60	very good

## Supplementary Materials

The following are available online at https://www.mdpi.com/2073-4360/12/5/1190/s1, Figure S1: The deconvolution of XRD patterns of pure PVA film.
