# Supplementary material for: Transparent Ultraviolet (UV)-Shielding Films Made from Waste Hemp Hurd and Polyvinyl Alcohol (PVA)
Source: Polymers (Basel). 2020 May 22;12(5):1190. doi: 10.3390/polym12051190 (PMC7284461; doi:10.3390/polym12051190)
Supplement: Supplementary file 1 [file polymers-12-01190-s001.pdf]

polymers

Article

Supplementary Materials

Transparent Ultraviolet (UV)-shielding films made from waste hemp hurd and Polyvinyl Alcohol (PVA)

Yi Zhang[1], Rechana Remadevi[1], Juan P Hinestroza[2], Xungai Wang[1], Maryam Naebe[1*]

[1] Deakin University, Institute for Frontier Materials, 75 Pigdons Road, Geelong, Victoria, 3216, Australia
[2] Cornell University, Fiber Science and Apparel Design, thaca, New York 14853, United States
* Correspondence: maryam.naebe@deakin.edu.au;

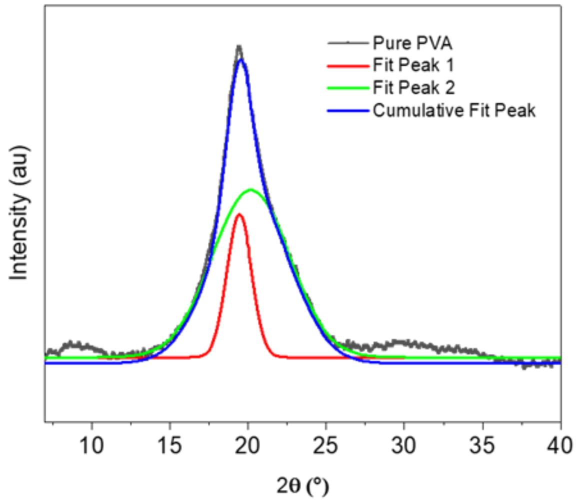

Figure S1. The deconvolution of XRD patterns of pure PVA film
